# Effect of Pharmaceutical Potential Endocrine Disruptor Compounds on Protein Disulfide Isomerase Reductase Activity Using Di-Eosin-Oxidized-Glutathion

**DOI:** 10.1371/journal.pone.0009507

**Published:** 2010-03-03

**Authors:** Danièle Klett, Claire Cahoreau, Mélanie Villeret, Yves Combarnous

**Affiliations:** Institut National de la Recherche Agronomique (INRA), Centre National de la Recherche Scientifique (CNRS), Unit « Physiologie de la Reproduction et des Comportements », Nouzilly, France; University of Southampton, United Kingdom

## Abstract

**Background:**

Protein Disulfide Isomerase (PDI) in the endoplasmic reticulum of all cells catalyzes the rearrangement of disulfide bridges during folding of membrane and secreted proteins. As PDI is also known to bind various molecules including hormones such as estradiol and thyroxin, we considered the hypothesis that adverse effects of endocrine-disrupter compounds (EDC) could be mediated through their interaction with PDI leading to defects in membrane or secreted proteins.

**Methodology/Principal Findings:**

Taking advantage of the recent description of the fluorescence self quenched substrate di-eosin-oxidized-glutathion (DiE-GSSG), we determined kinetically the effects of various potential pharmaceutical EDCs on the *in-vitro* reductase activity of bovine liver PDI by measuring the fluorescence of the reaction product (E-GSH). Our data show that estrogens (ethynylestradiol and bisphenol-A) as well as indomethacin exert an inhibition whereas medroxyprogesteroneacetate and nortestosterone exert a potentiation of bovine PDI reductase activity.

**Conclusions:**

The present data indicate that the tested EDCs could not only affect endocrine target cells through nuclear receptors as previously shown, but could also affect these and all other cells by positively or negatively affecting PDI activity. The substrate DiE-GSSG has been demonstrated to be a convenient substrate to measure PDI reductase activity in the presence of various potential EDCs. It will certainely be usefull for the screening of potential effect of all kinds of chemicals on PDI reductase activity.

## Introduction

Endocrine-disrupting compounds are commonly considered as molecules acting either by mimicking or by blocking the transcriptionnal activation of hormone nuclear receptors such as those for estrogens, androgens, progestagens, thyroid hormones etc [1], [2]. Nevertheless, other pathways are now taken into consideration because these hormones can affect nuclear receptors indirectly [3] or act through non-genomic pathways [4] or can bind to non-receptor cellular proteins [5].

In the frame of the european *Food* & *Fecundity* project (http://foodandfecundity.factlink.net/180095.1/), one of the main strategic objectives was to identify pharmaceutical products as potential endocrine disruptors of reproductive function and to determine their adverse effects and mechanisms of action in *in-vitro* and *in-vivo* systems. A prioritization list of seven pharmaceutical products bearing a potential of affecting human fecundity by entering the food chain has been created on the basis of production volume, recognized presence in environment and documented effect(s) on reproduction [6]. The main drug of concern in this respect was 17α-ethinylestradiol that is the estrogenic component of most contraceptive pills.

Besides, it has been reported that Protein Disulfide Isomerase which catalyzes oxidative folding of proteins [7] in the endoplasmic reticulum has also been found to be a high-capacity binding protein for 17β-estradiol [5]. It was also found by the same authors that 17β-estradiol displayed an inhibitory effect on isomerase activity of PDI using scrambled RNA as substrate [5]. Taking this information into account, we considered the hypothesis that EDCs could exert part of their effects by affecting PDI properties. We have thus undertaken a study of the influence of estrogens as well as other pharmaceuticals of interest on another enzymatic function of PDI, i.e. its disulfide reductase activity.

## Results

Preliminary control experiments were carried out in order to check whether reduction of DiE-GSSG by another enzyme than PDI or its cleavage by various proteases or conformational changes could interfere in the assay (see figure S1 in supplementary data). We found that on a molar basis, thioredoxin exhibited less than 0.1% reductase activity on DiE-GSSG compared to PDI. Likewise, Glutathion Reductase either in the presence of 1 mM NADPH or 33 µM DTeT was unable to reduce DiE-GSSG. Several proteolytic enzymes (trypsin, thrombin, collagenase-dispase, leucine-aminopeptidase and carboxypeptidase) were tested and found to be without any effect on DiE-GSSG fluorescence. Moreover, neither 0.1% Tween nor 0.5 M concentrations of sodium chloride or ammonium sulfate had any direct effect on DiE-GSSG fluorescence. All these negative controls indicate that the reductase assay using DiE-GSSG as a substrate is highly specific for PDI (see supplementary material).

**Figure 1:**
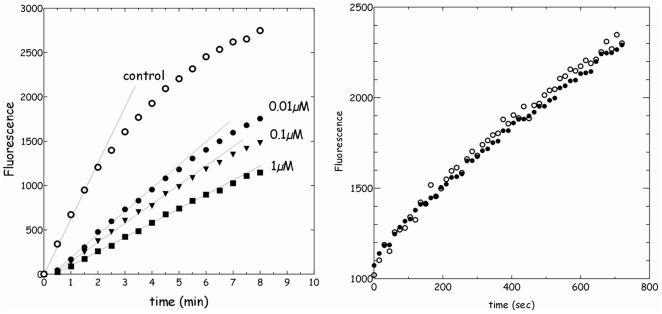
PDI-catalyzed reduction of di-eosin oxidized-glutathion (DiE-GSSG) in the absence or presence of increasing concentrations of ethynylestradiol (EE2). **A/** Kinetics of PDI-catalyzed reduction of DiE-GSSG in the absence or presence of increasing concentrations of ethynylestradiol (EE2). PDI activity is determined using initial velocity of the reaction as measured through the abolishment of fluorescence self quenching in 2.4 µM DiE-GSSG when it is reduced into two molecules of E-GSH in the presence of 33 µM DTeT. **B/** Comparison of Kinetics of PDI-catalyzed reduction of DiE-GSSG as in A/ in the absence (○) or presence (•) of 10% (v/v) ethanol in the 100 mM phosphate buffer pH 8.0.

**Figure 2:**
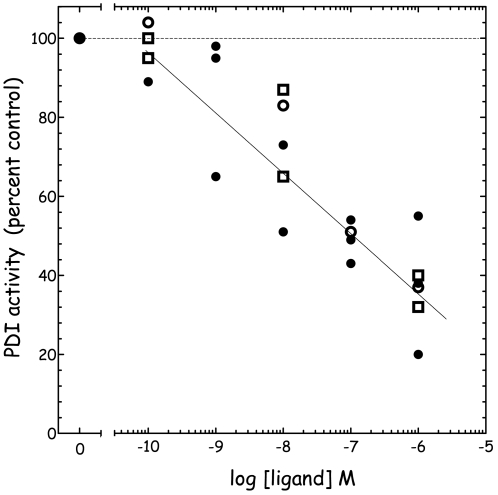
Dose-dependent inhibition of PDI reductase activity by the estrogenic compounds E2 (○), DES (•) and EE2 (□). PDI activities represent initial velocities determined by kinetics of DiE-GSSG reduction as described in fig. 1.

Figure 1A shows the kinetics of DiE-GSSG reduction catalyzed by bovine liver PDI. A dose-dependent inhibition of PDI by EE2 can be evidenced through the measurement of the initial rate of the reaction. Figure 1B shows that the kinetics of DiEGSSG reduction by PDI as followed by EGSH fluorescence is insensitive to the presence of ethanol up to 10% (v/v). In figure 1A, the maximum ethanol concentration (for EE2 1 µM) is less than 0.01%. The estrogens E2 and DES exhibited similar effects as EE2 as shown in figure 2. Indeed, figure 2 reports the effects of increasing concentrations of the various estrogenic EDCs under study (E2, EE2, DES) on the initial velocity of PDI activity using DiE-GSSG and DTeT as co-substrates. Interestingly, the three estrogenic molecules exhibit a significant inhibitory effect on PDI activity starting at concentrations as low as 10^−8^ M. BPA was also found to exert a partial (20%) inhibitory effect on PDI reductase activity but only at concentrations higher than10^−4^ M (not shown).

**Figure 3:**
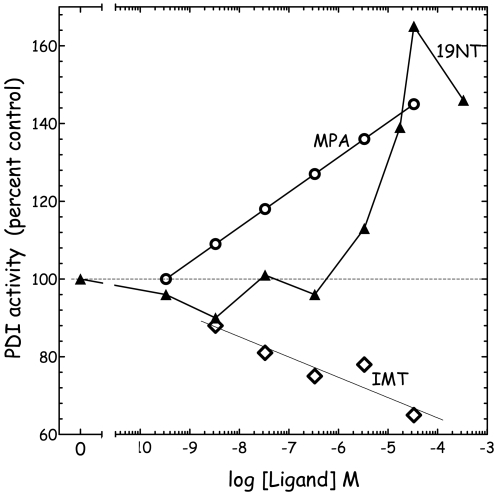
Dose-dependent effect of MPA (○), 19-NT (▴) and IMT(⋄) on PDI reductase activity. PDI activities represent initial velocities determined by kinetics of DiE-GSSG reduction as described in fig. 1.

Figure 3 shows the effects of three non-estrogenic potential EDCs, MPA, 19-NT and IMT, on PDI activity. Only IMT exhibits an inhibitory effect which is however much less important than that of estrogenic molecules (fig. 2). By contrast, the two non-estrogenic steroids MPA and 19-NT exhibit a potentiation effect on PDI reductase activity but only above 10^−5^ M for 19NT.

In addition we checked that 10^−4^ M bacitracin that is a known inhibitor of PDI activity [8], [9] also exhibited such an inhibitory activity in this protocol (not shown).

## Discussion

Protein Disulfide Isomerase (PDI) is a multifunctional enzyme mainly found in the endoplasmic reticulum of eukaryotes where its main function is to catalyze the rearrangement (isomerization) of disulfide bridges during folding of membrane and secreted proteins. Its concentration in the lumen of the endoplasmic reticulum is known to be very high [10] and it has been reported to act as a high capacity reservoir for various ligands including hormones such as estradiol (E2) and thyroxine (T3) [5], [11]. This ability of PDI to bind a large number of different molecules prompted us to search whether potential encocrine disruptor compounds (EDCs) could exert an influence on its reductase activity. For this matter, we used the recently described fluorescence self-quenched substrate DiE-GSSG [12] that is easier to synthesize and exhibits a much higher signal than the previously described substrate di-aminobenzoyl-GSSG [13].

Since eosin fluorescence in DiE-GSSG is quenched because of the vicinity of eosin-goups at the N-termini of oxidized glutathion (GSSG), it was important to check that reduction by another enzyme than PDI or proteolysis or conformational change of GSSG could not interfere in the assay. Thioredoxin exerted a very marginal DiE-GSSG reducing activity (0.1%) compared to PDI. This observation indicates that the b and b' domains of PDI [14] play an important role in addition to the thioredoxin-like a and a' domains to permit efficient reducing activity on DiE-GSSG. Glutathion Reductase was found to be devoid of activity whatever reducing co-substrate was used (1 mM NADPH or 33 µM DteT). None of the tested or proteases provoked any change in DiE-GSSG fluorescence. All these data indicate that DiE-GSSG is a highly specific substrate for the measurement of PDI reductase activity.

The most interesting observation was that the three estrogenic molecules that have been tested (E2, EE2 and DES) exerted an inhibitory effect on PDI reductase activity. This inhibition was observed with concentrations as low as 1–10 nM. These very low concentrations were unexpected as a Kd of 2.1 µM had been previously determined for the binding of E2 to rat PDI [5]. A possible origin of the observed discrepancy is that these authors studied recombinant rat PDI expressed in E. coli [[15], [16]] whereas bovine liver PDI was used in the present study. It can be suspected that the redox potential in endoplamic reticulum environment, less reductive than that in bacteria, is of utmost importance for the correct folding of PDI and consequently recombinant PDI expressed in E.coli might be unappropriate.

An inhibitory effect of micromolar concentrations of E2 on the isomerase activity of the recombinant rat PDI in the RNA refolding assay had been observed [5]. In the present work, we followed the reductase activity of bovine liver PDI instead of its isomerase activity. Therefore, it could also be that estrogens exert their effects on these two activities of PDIs at different concentrations. Nevertheless, in both cases estrogens have been shown to interact with PDI and to inhibit its activities. Numerous EDC affecting reproduction are estrogenic molecules such as EE2, DES, BP-A etc… [17]–[19]. The observation of diminished PDI reductase activity in the presence of physiological concentrations of estrogens indicates that these hormones might have an inhibitory effect on the synthesis and secretion of proteins including by cells not expressing estrogen receptors. It must also be taken into consideration that PDI can be found at the surface of plasma membrane where it is thought to exert various functions [20]. Circulating estrogens could thus be expected to play a role at this level.

The data in the present paper also show that non-estrogenic steroids such as the progestagen MPA and the androgen 19NT not only do not exhibit any inhibiting effect on PDI reductase activity but, in contrast, exert unexpected potentiation effects.

Previous papers have reported interaction of PDI with estrogenic molecules such as estradiol [5], [21]–[23] or bisphenol-A [24], [25]. It has also been shown to interact with other hormones such as T3 [11] and many other molecules. In the present report, we have focused on the *in vitro* effects of potential endocrine disrupters of reproduction on the reductase activity of PDI.

Endocrine disruptor compounds (EDC) are suspected to affect human and animal health with possible consequences for development and evolution. Indeed, they act either through hormone receptors, particularly nuclear receptors [26] or epigenetically through various mechanisms [4]. Considering the large amount of PDI in all cells as well as its crucial role in the correct folding of membrane and secreted proteins in the endoplasmic reticulum, it is of concern that all the EDCs tested in present work exhibited negative (estrogens, indomethacin) or positive (progestagen, androgen) effects on its reductase activity. These data strongly suggest that EDCs could affect not only endocrine target cells through nuclear receptors but also all cells through PDI and consequently by affecting folding of proteins in the endoplasmic reticulum. Defective protein folding has been described as a basis for human disease [4], [27]–[29] and should thus be taken into consideration in the study of detrimental effects of EDCs.

Extrapolation of the present data to *in vivo* exposures is obviously difficult. Obviously, each individual potential EDC tested in the present study is not expected to reach a sufficient concentration *in vivo* to exert by itself a significant effect on PDI activity. Nevertheless, owing to the large number of molecules able to interact with PDI, additive or even synergistic effects can be envisionned and could lead to significant *in vivo* effects.

The availability of the new substrate PDI DiE-GSSG [12] offers a rapid and easy way to test all suspected molecules for their eventual effect on PDI activity. Since neither Thioredoxin nor Glutathion Reductase reduce DiE-GSSG in the presence of NADPH or DTeT, this substrate appears to be highly specific for PDI. This assay should thus be used as a screen to detect molecules that, individually or synergisticallly, would be potentially harmful for cell function by affecting protein folding.

## Materials and Methods

17β-estradiol (E2), 17α-ethinylestradiol (EE2), diethylstilbestrol (DES), medroxyprogesterone acetate (MPA), 19-nor-testosterone (19NT), bacitracin, indomethacin (IMT), bisphenol A (BPA), dithioerythreitol (DTeT), eosin 5-isothiocyanate, oxidized glutathion (GSSG), Protein Disulfide Isomerase (E.C. 5.3.4.1) from bovine liver (PDI), Thioredoxin (E.C 1.6.4.5) from spirulina and Glutahtion Reductase (E.C 1.6.4.2) from S. cerevisiae as well as the proteolytic enzymes trypsin, thrombin, collagenase-dispase, leucine-aminopeptidase and carboxypeptidase were all purchased from Sigma-Aldrich (Isle-d'Abeau, France) and were of the highest available grades. All steroids were initially disolved in ethanol and serially diluted in the 100 mM phosphate buffer pH 8.0 used for the kinetics.

The PDI substrate, di-eosin-oxidized glutathion (diE-GSSG), was synthesized and purified as previously described [12] with minor modifications. After final purification on sephadex G50 (Pharmacia, Uppsala, Sweden) in water, aliquots of eluted DiE-GSSG were freeze-dried and kept in the dark until use.

PDI reductase activity was measured through abolishment of fluorescent self quenching when DiE-GSSG is reduced into two molecules of E-GSH. Initial velocities in fluorescence increase (λ_exc_ = 518 nm; λ_em_ = 545 nm) were recorded using a Spectra-Max Gemini spectrofluorimeter (Molecular Devices, Sunnyvale, CAL USA) and analyzed with SoftMaxPro program. Concentrations of the reagents at t_0_ were 200–333 nM for PDI, 2.4 µM for DiE-GSSG, 33 µM for DTeT and 0.1 nM to 100 µM for the various molecules under study (E2, EE2, DES, MPA, 19NT and IMT).

## Supporting Information

File S1 Control experiments showing the effects of reductive enzymes and proteolytic enzymes on DiE-GSSG fluorescence (λ_exc_ = 518 nm; λ_em_ = 545 nm)
(0.19 MB DOC)
