# Supplementary material for: Effect of Pharmaceutical Potential Endocrine Disruptor Compounds on Protein Disulfide Isomerase Reductase Activity Using Di-Eosin-Oxidized-Glutathion
Source: PLoS One. 2010 Mar 3;5(3):e9507. doi: 10.1371/journal.pone.0009507 (PMC2831067; doi:10.1371/journal.pone.0009507)

Supplementary data

Control experiments showing the effects of reductive enzymes and proteolytic enzymes on DiE-GSSG fluorescence (λ_exc_ = 518 nm; λ_em_ = 545nm)

Abolition of quenching in Die-GSSG fluorescence can be potentially due either to reduction of its disulfide bond or to hydrolysis of its peptide bond between the N-terminal Glu and cystine residues. As controls, we therefore tested several enzymes with reductase activity and proteolytic enzymes.

**Figure S1** : **Kinetics of of di-eosin oxidized-glutathion (DiE-GSSG; 2.4µM) fluorescence quenching abolition in the presence of various enzymes** :

Bovine PDI (0.2µM with 33µM DTeT ), yeast Glutathion Reductase (0.3 and 3 µM with 1mM NADPH), thioredoxin (40µM with 33µM DteT), trypsin, thrombin, leucine-aminopeptidase (3 µM).

Carboxypeptidase and collagenase were also tested but are not shown for the sake of clarity as they exhibited no activity like trypsin, thrombin and leucine-aminopeptidase.


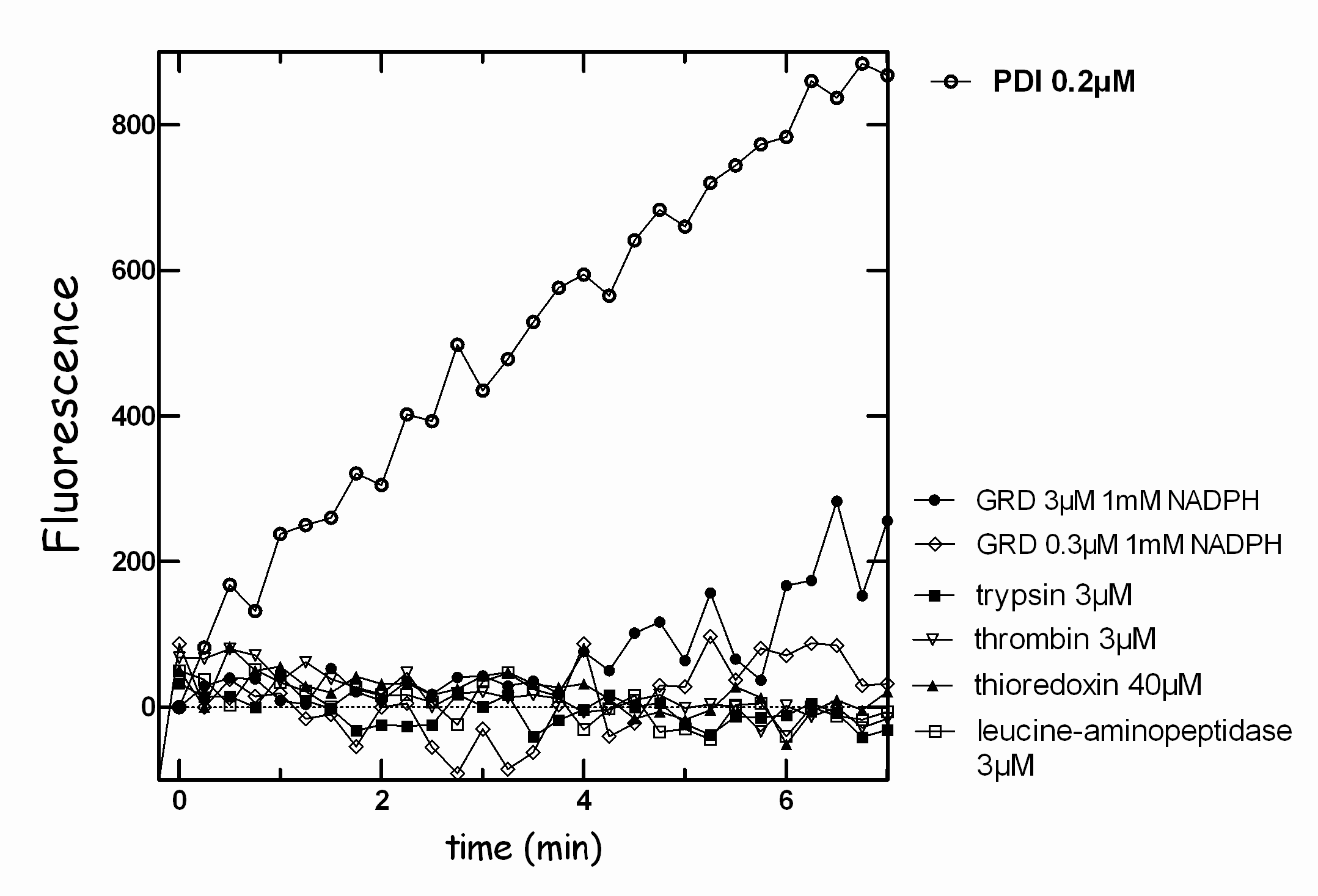

Supplement: File S1 — Control experiments showing the effects of reductive enzymes and proteolytic enzymes on DiE-GSSG fluorescence (λexc = 518 nm; λem = 545 nm) (0.19 MB DOC) [file pone.0009507.s001.doc]
